# Emergency Department Opt‐Out Testing for Hepatitis B: A Two‐Year UK Multicentre Evaluation of Outcomes Across Seven Sites

**DOI:** 10.1111/liv.70665

**Published:** 2026-04-30

**Authors:** Jennifer J. Plunkett, Basil Ahmad, Amy Teague, Tanzina Haque, Stephanie Paget, Jennifer Hart, Maximillian Habibi, Jonathan Durban, Shiny Jaimes, Sally Thomas, Helen Boothman, Supa Chantschool, Philippa C. Matthews, Rachel Hill‐Tout, Daniel Forton, Douglas Macdonald

**Affiliations:** ^1^ Department of Hepatology Royal Free Hospital, Royal Free London NHS Trust London UK; ^2^ Department of Gastroenterology and Hepatology St George's University Hospitals NHS Foundation Trust London UK; ^3^ Department of Virology Royal Free London NHS Trust London UK; ^4^ Department of Clinical Infection St George's University Hospitals NHS Foundation Trust London UK; ^5^ HepB Companion London UK; ^6^ The Francis Crick Institute London UK; ^7^ Division of Infection and Immunity University College London London UK; ^8^ Department of Infectious Diseases University College London Hospital London UK; ^9^ Mortimer Market Centre Central Northwest London NHS Trust London UK; ^10^ NHS England London UK; ^11^ Institute for Infection and Immunity St George's, University of London London UK; ^12^ Institute for Liver and Digestive Health UCL, Hepatology, Royal Free London NHS Trust London UK

## Abstract

**Background and Aims:**

An estimated 270 000 people in the UK live with hepatitis B infection, the leading global cause of liver cancer. In 2022, opt‐out hepatitis B testing was introduced in emergency departments (ED) in London. We conducted a 2‐year multicentre evaluation of this programme across seven sites.

**Methods:**

Adults testing positive for hepatitis B surface antigen (HBsAg) through ED opt‐out testing (*n* = 983) were compared with those referred via non‐ED pathways (*n* = 416) over at least 12 months in the same regions with a six‐month follow‐up period. Demographics, clinical characteristics and factors influencing time to assessment were analysed.

**Results:**

ED testing led to a 107% increase in HBV assessments. Of 983 HBsAg‐positive individuals, 90.2% (887/983) were contactable and 97% (660/679) of those requiring assessment were linked to care. 35% were aware of their diagnosis but not under specialist care. Among ED‐diagnosed individuals, 16.37% had significant fibrosis and 20.45% had viral loads > 2000 IU/mL. ED referrals were older (mean age 51 vs. 47 years, *p* < 0.001) and had lower viral loads (mean log10 HBV DNA 2.08 vs. 2.78, *p* < 0.001). Mean time to assessment in the ED group was 90 days.

**Conclusions:**

ED opt‐out testing has doubled new assessments of hepatitis B cases, identifying individuals who would benefit from surveillance and/or treatment. Linkage‐to‐care rates were very high, though time to assessment was prolonged by service factors. A significant proportion were aware of their diagnosis but lost to care, underscoring the need for services which can maintain engagement.

## Lay Summary

This study evaluated routine opt‐out hepatitis B testing in seven UK emergency departments, where patients were tested automatically unless they declined. In a matched time period, this programme more than doubled the number of hepatitis B assessments taking place compared with traditional referral routes and identified many people who were previously unaware of their diagnosis or had lost contact with care. The majority of those needing specialist follow‐up were linked to services, showing that emergency department opt‐out testing is an effective way to find new cases and engage people living with hepatitis B in long‐term care.

## 1 Introduction

Hepatitis B virus (HBV) chronic infection contributes significantly to the global burden of liver cirrhosis and is the leading cause of death from liver cancer world‐wide [1]. The World Health Organisation (WHO) estimates that there were 254 million people living with hepatitis B (PLWHB) infection in 2022, with 1.2 million new infections each year [2]. There were an estimated 1.3 million deaths related to hepatitis B globally in 2022 and this number is rising unlike mortality from other communicable diseases [2]. Suppression of viral replication can be achieved with nucleo(t)side analogue (NA) reverse transcriptase inhibitors, which improve survival, reduce progression to cirrhosis, lower risk of hepatocellular cancer (HCC) and reduce transmission [3, 4, 5]. Prevention of transmission with vaccination of at‐risk contacts and neonates is also essential for reducing incidence.

The WHO have set specific targets for the elimination of hepatitis B infection as a public health threat by 2030. These include reducing the undiagnosed population to < 10% of PLWHB and ensuring at least 80% of those eligible are commenced on treatment [6]. In the UK it is estimated there are 270 000 PLWHB (prevalence 0.6%) with more than half undiagnosed [7]. Barriers to testing and clinical care include lack of awareness, stigma and constrained resources [8, 9]. It is estimated that 95% of PLWHB in the UK are migrants who experience multiple specific barriers to accessing healthcare [7].

The UK hepatitis C elimination programme began in 2019 and has led to innovative case‐finding and engagement strategies which can be adapted for other aetiologies of liver disease. Targeted testing and micro‐elimination strategies for hepatitis C have been successful in the UK among the prison population and within drug and alcohol services [10, 11, 12]. Co‐ordinated testing initiatives for hepatitis B, however, have not been deployed beyond maternal antenatal screening, which was introduced in 2000 in the UK, with routine vaccination for neonates following in 2017 [7, 13].

In 2022, the National Health Service England (NHSE) commissioned a staged roll‐out of blood‐borne virus (HIV, HBV and HCV) opt‐out testing in selected emergency departments (ED). Several pilot studies were carried out in the years prior showing feasibility and cost effectiveness on a smaller scale [14, 15, 16, 17]. The roll‐out began in London EDs where HIV opt‐out testing had already been piloted and prevalence was known to be high. It was posited that the HBV prevalence would also be elevated in these areas justifying inclusion in combined blood‐borne virus (BBV) testing. This has since been confirmed by the ED opt‐out testing interim evaluations and several London‐based studies of the first two years of the programme. 1957 new hepatitis B diagnoses, 762 new hepatitis C diagnoses and 391 new HIV diagnoses were reported across 21 ED testing sites between April 2022 and March 2024 [18]. Various London‐based studies have shown a prevalence of for hepatitis B infection in the ED population to be 0.58%–0.89% and linkage to care rates of 57%–78.9% [19, 20, 21, 22, 23].

The absence of a large‐scale national dataset encompassing hepatitis B infection assessments and treatments means that the UK Health Security Agency (UKHSA) is unable to measure the numbers of patients benefitting from linkage to care from ED testing, nor can the characteristics of this cohort be compared with those referred via standard pre‐existing routes into hospital‐based specialist care (e.g., antenatal screening, reactive testing after abnormal liver function tests and NICE guideline opportunistic targeted screening in primary care). We therefore undertook a two‐year multicentre retrospective evaluation of referrals to hepatitis services through ED opt‐out testing, compared to standard testing and referral routes (‘non‐ED’) over the same period. We examined demographics and clinical characteristics, linkage to care and factors affecting time to first assessment with specialist services.

## 2 Methods

**FIGURE 1:**
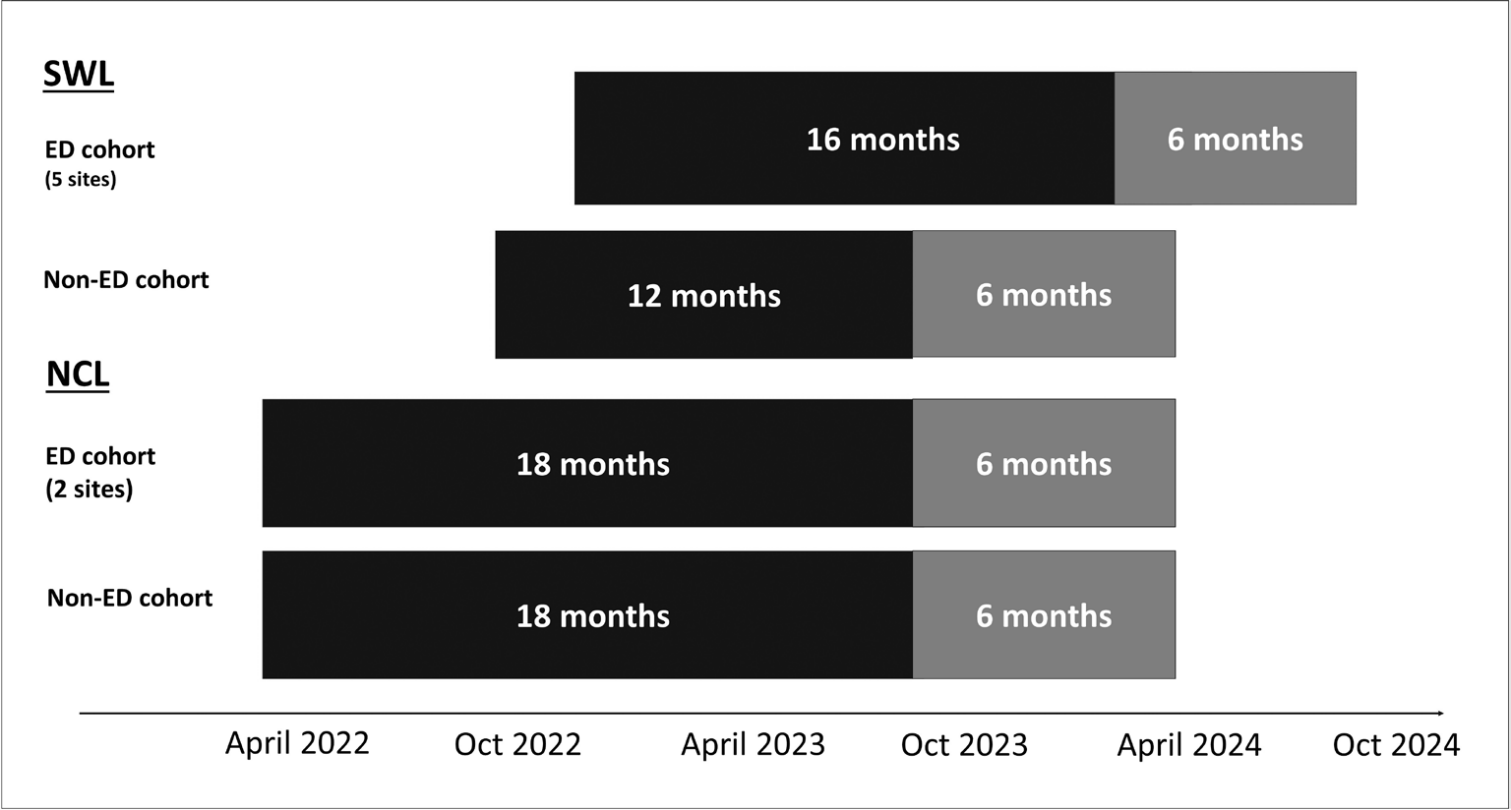
Timeline of cohort evaluations for ED and non‐ED HBsAg positive individuals in North Central London (NCL) and Southwest London (SWL). ED opt‐out testing was introduced later in SWL; the ‘non‐ED’ cohort were recruited over a shorter time period due to data availability. In both ‘non‐ED’ cohorts all referrals were received and assessed at the hub sites for each region.

The London ED screening programme was rolled out from April 2022 by NHSE as part of routine clinical care in participating centres. Any individual over 18 who presented to ED and required a blood test was also tested for HIV, HCV and HBV unless they opted out. Posters and leaflets were made available in the ED to inform patients of the new initiative. Data from seven London EDs are reported here; two sites in the North Central London (NCL) region and five in Southwest London (SWL). The ED sites in both regions serve a total of 2.5 million people, a large representative population of London. One hospital site in each region with a dedicated viral hepatitis service (‘hub site’) provides notification, coordination and assessment of patients diagnosed at all other hospital ED sites in the region. At the two ED sites in NCL, individuals diagnosed with hepatitis B via ED testing were included over 18 months from April 2022 until October 2023. At the five ED sites in SWL, individuals diagnosed via ED testing were included over 16 months from November 2022 until March 2024 (the programme began later in this region). There was a six‐month follow up period for each cohort to allow time for assessment and engagement in services (Figure 1).

Individuals who did not opt out were tested in ED for HBsAg, HCV antibody and HIV antibody. All virological testing was performed by UK accredited services as described previously [19, 20]. In NCL index reactive HbsAg samples were reflex tested for anti‐HBc total, anti‐HBc‐IgM, anti‐HBe/HBeAg and HDV antibody. In SWL patients these were reflexed tested on a subsequent confirmatory sample and HDV antibody was directly requested (non‐reflex) by the virology team. In both regions HBV DNA was directly requested by the virology team on index (NCL) or confirmatory (SWL) samples with reactive HBsAg. The virology team reported HBsAg positive and equivocal results to the hepatology clinical nurse specialist (CNS) team in the coordinating hub for each region. These teams were responsible for contacting patients by phone to inform them of the diagnosis and arrange an assessment at the hub site. If an individual was not contactable after three phone call attempts, a letter was sent to them and to their GP to inform them of the test result and invite them for assessment.

A comparison cohort of patients with hepatitis B infection referred from ‘non‐ED’ routes to coordinating hubs was included over 18 months for NCL sites and over 12 months in SWL. Changes in referral systems and data collection thereof in SWL required limiting the recruitment window for non‐ED assessments to 12 months with an overlapping window for comparison with ED assessment volumes to 9 months (Figure 1). Non‐ED routes of referral included primary care, secondary care, antenatal clinics as well as community and outreach services. Testing for HBV may have been offered for a variety of reasons in these settings including investigation of abnormal liver function tests, abnormal liver imaging and/or risk factors for infection. Individuals diagnosed after testing in primary care are referred to the specialist viral hepatitis team via an electronic referral system, whilst individuals diagnosed in other settings may be referred by directly contacting the viral hepatitis team. Referrals are triaged by a clinician in terms of urgency and booked into a clinic by patient co‐ordinators. In both NCL and SWT regions, all ‘non‐ED’ referrals were received and assessed in specialist clinics at the hub site in the same manner as ED testing referral. Both ‘non‐ED’ cohorts represent the same geographical area and population as the ‘ED’ cohorts.

Demographics and clinical information were recorded where available for every individual in ED and non‐ED cohorts using electronic patient records at each hospital site. Ethnicity and first language were self‐reported on arrival to the ED in some patients. Additional demographic data and assessment of liver disease status (Fibroscan liver stiffness measurement (LSM), controlled attenuation parameter (CAP)) were collected and recorded on electronic patient records at the first viral hepatitis clinic attendance. Progression to treatment was defined as commencing a nucleo(t)side analogue within the censor dates. Data were stored on an encrypted spreadsheet. Where ethnicity data were available these were categorised into Office for National Statistics (ONS) 8a categories [24].

Table 1 shows the study questions, the comparison cohorts for each, the parameters analysed and the statistical tests used. Data were analysed with IBM SPSS v29 software. In addressing question 1 (‘What was the impact of ED testing on numbers of patients assessed’) cohorts were limited to exactly matched overlapping referral time windows and sites in both NCL and SWL regions and thus have slightly smaller numbers than subsequent comparison cohorts. For questions 2 and 3 cohort numbers differed due to the availability of the compared parameters between referred and assessed groups (with demographics being available in the both but disease‐related parameters only being available in those progressing to assessment).

**TABLE 1:** Overview of study questions, cohort definitions, assessed parameters and analysis performed.

Question	Cohort 1 (*n*)	Cohort 2 (*n*)	Parameter	Analysis
1. What was the impact of ED testing on numbers of patients assessed?	ED referrals assessed within overlapping referral time window and location (*n* = 405)	Non‐ED referrals assessed (*n* = 377)	Total number assessed	Descriptive
2. Did ED and non‐ED cohorts differ in demographics?	ED referrals (*n* = 774)	Non‐ED referrals (*n* = 416)	Age, gender, ethnicity, country of birth, language	Univariate *t*‐tests of normally distributed (or log normalised) data, Chi‐squared tests of proportions (with Fisher's exact test where appropriate) Binary logistic regression was used for multivariate analysis
3. Did ED and non‐ED cohorts differ in viral and liver indices of disease?	ED referrals assessed (*n* = 660)	Non‐ED referrals assessed (*n* = 394)	_Log_(LSM), CAP, Proportions ≥ F2, Proportions ≥ F4 _Log_(HBV DNA), proportion HBV DNA > 2000/> 20 000 IU/mL, proportions initiating treatment	
4. What factors influenced time to assessment in the ED cohort?	ED referrals (*n* = 774)		Age, gender, alcohol use, UK‐born, English as a first language, previous awareness of diagnosis, planned assessment at the same site of diagnosis	Cox regression multivariate analysis

*Note:* The total individuals used in each analysis (*n*) differs depending on whether the analysis was performed on a cohort of all referrals or those who were linked to care (assessed). Analysis of question 1 was performed on an ‘ED’ and ‘non‐ED’ cohort assessed across the same locations; the recruitment period was shortened in both SWL cohorts to ensure an overlapping time‐window. This has resulted in a smaller (*n*) number in both groups.

## 3 Results

**FIGURE 2:**
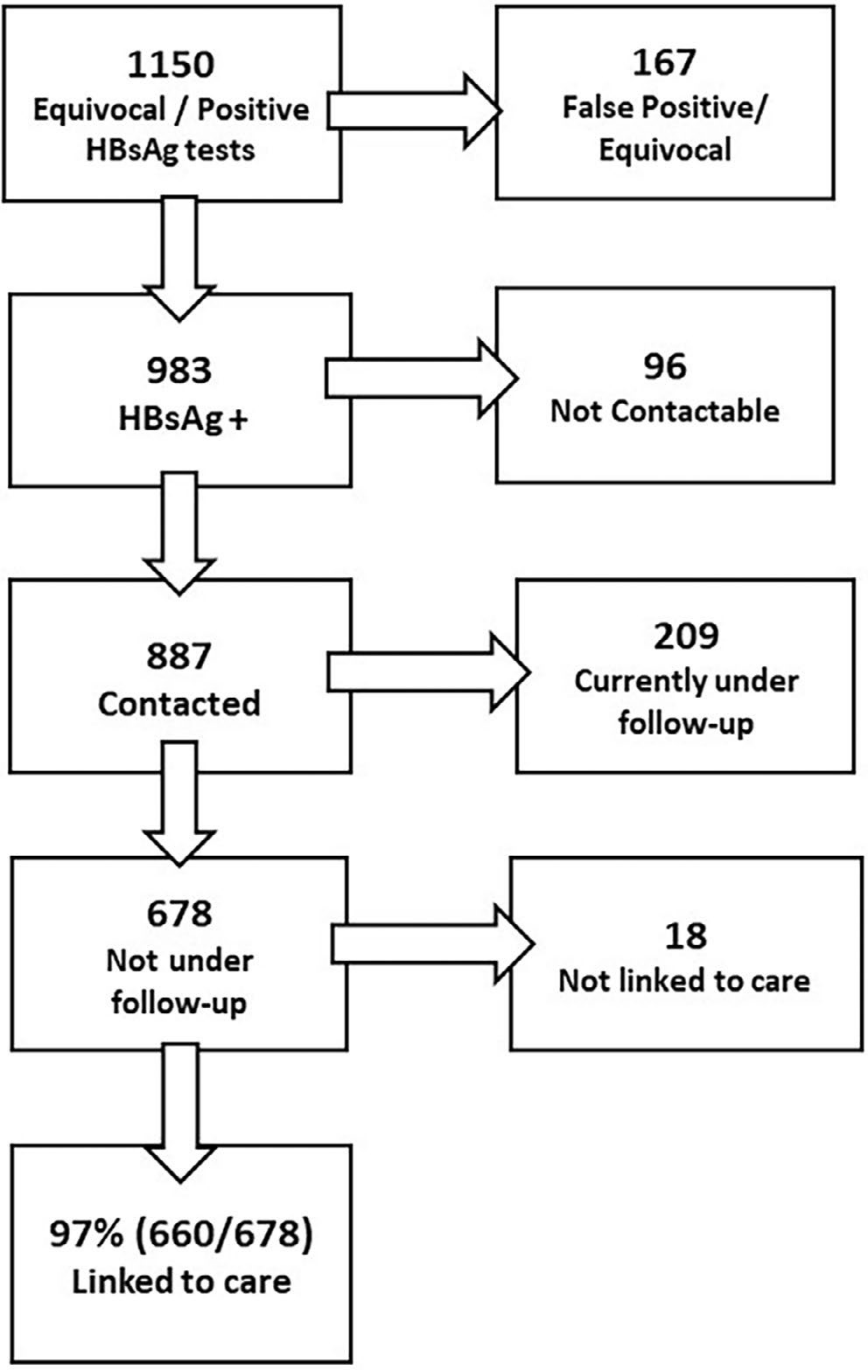
Flowchart from diagnosis to assessment in adults testing HBsAg‐positive or equivocal through ED testing across 7 sites.

339 627 HBsAg tests were performed at the seven ED sites between April 2022 and March 2024. These represent 29% of all tests performed in the same period of the national ED testing programme. There were 983 HBsAg positive results notified by the virology team within the study period (Figure 2). Additionally, there were 169 equivocal HBsAg results, two of which were positive on repeat testing and the remainder negative (142) or still awaiting retesting (25). The two individuals who were initially HBsAg equivocal then subsequently positive on retesting have been included in the 983 HBsAg individuals shown in Figure 2. Of the 983 HBsAg positive individuals, 90% (887/983) were contactable either by phone or after notification by letter. Of these, 60.2% (534/887) were new diagnoses not previously known to services, 16.2% (144/887) were lost to follow‐up and 23.6% (209/887) were already under follow‐up, either locally or elsewhere.

Of those who were contactable and required linkage to care, 97% (660/678) were successfully assessed in specialist hub clinics. Including those who were not contactable (*n* = 96), 85% (660/774) were successfully linked to care within the censor dates (though it cannot be ascertained what proportion of those not contactable were under care elsewhere). Of those assessed in specialist clinics, 35% (231/660) were already aware of their diagnosis; this included 91 individuals who had never been under specialist care. In a matched recruitment window, to allow for a shorter observation period in the SWL ‘non‐ED’ cohort, 405 patients diagnosed via the ED testing route were compared to 377 referred via standard routes (‘non‐ED’). In NCL, 18 months of ED opt‐out testing led to a 113% (307/271) increase in assessments for hepatitis B infection. In SWL from November 2022 to September 2023, ED testing led to a 92.5% (98/106) increase in assessments for hepatitis B infection. Overall, this represents a 107% increase in assessments above those from non‐ED referral sources.

Across all ED testing new referrals not under follow‐up (*n* = 774) the mean age was 51 years (SD = 14.5); two thirds (60.2%) of these individuals were male. In the subset of assessed individuals, all had a documented HBV DNA level and 94.4% (623/660) had a non‐invasive liver stiffness measurement (LSM) using Fibroscan. Of these, 16.37% (102/624) had an LSM ≥ 7.0 kPa, indicating the presence of at least F2 fibrosis and would likely benefit from treatment and HCC surveillance [23, 24]. Additionally, 2.1% (13/624) had an LSM in the cirrhotic range (≥ 12.5 kPa), the majority of whom (11 of 13) had never previously been assessed in clinic. In total, 20.45% (135/660) of individuals had a baseline HBV DNA > 2000 IU/mL and 7.88% (52/660) had HBV DNA > 20 000 IU/mL.

Both LSM ≥ 7.0 kPa and viral load > 2000 IU/mL are indications for treatment eligibility under new WHO guidance [25]. During the evaluation period, 14.39% (95/660) of assessed individuals progressed to treatment. Two individuals with hepatocellular carcinoma (HCC) were identified, both of which were confirmed with subsequent liver biopsy. Seven individuals with active hepatitis D (HDV) co‐infection were found in the ED group.

Comparisons of demographic, clinical and laboratory characteristics between ED and non‐ED cohorts are shown in Table 2. The ED cohort was older (mean age 51 years (ED) vs. 47 years (non‐ED), 2‐sided *p* value < 0.001). There was no difference in gender, English‐speaking status or the proportion of patients born in the UK between both cohorts (all 2‐sided *t*‐tests, NS). There was a higher proportion of individuals identifying as Black British, African or Caribbean in the ED group (37.6% ED vs. 24.4% non‐ED, 2‐sided *p* value < 0.001) and a higher proportion of individuals identifying as Asian or Asian British in the non‐ED cohort (19.9% ED vs. 33.4% non‐ED, 2‐sided *p* value < 0.001). The ED cohort included adults from 79 different countries of birth with 46 primary languages spoken. Although our country of birth data was incomplete, 94% (656/697) of patients in the ED cohort with a documented country of birth originate from countries with medium or high prevalence of HBV, recognised by national guidance (National Institute for Clinical Excellence, NICE PH43 2012) as an at‐risk group who should be offered targeted opportunistic testing in the course of routine care.

**TABLE 2:** Univariate and multivariate analyses comparing demographics and clinical characteristics in the ED and non‐ED cohorts across 7 sites.

Variable	Total (*n*) referrals: ED (*n* = 774), non‐ED (*n* = 416)	ED cohort	Non‐ED cohort	Test	df	Sig (2‐sided *p* value)
Demographics						
Age (years)	ED: *n* = 774 Non‐ED: *n* = 412	50.91 (SD = 14.52)	46.98 (SD = 13.8)	Independent *t*‐test	1184	< 0.001 (mean difference 3.93)
Gender	ED: *n* = 772 Non‐ED: *n* = 413	Male: 60.23% Female: 39.73%	Male: 60.05% Female: 39.95%	Pearson Chi square	1	0.95
English as 1st language	ED: *n* = 745 Non‐ED: *n* = 280	7.25% (*n* = 54)	6.87% (*n* = 18)	Fisher's exact test	1	0.78
UK‐born	ED: *n* = 653 Non‐ED: *n* = 244	3.83% (*n* = 25)	3.83% (*n* = 9)	Fisher's exact test	1	1.00
Ethnicity	ED: *n* = 667 Non‐ED: *n* = 338 ONS 8a categories [24]: 1: Asian 2: Black 3: Mixed 4: White British 5: White Irish 6: White Other 7: Other	Group 1—19.94% (*n* = 113)	Group 1—33.43% (*n* = 133)	Pearson Chi square	1	< 0.001
		Group 2—37.63% (*n* = 251)	Group 2—24.36% (*n* = 82)	Pearson Chi square	1	< 0.001
		Group 3—0.75% (*n* = 5)	Group 3—4.14% (*n* = 14)	Fisher's exact test	1	< 0.001
		Group 4—3.75% (*n* = 25)	Group 4—2.96% (*n* = 10)	Fisher's exact test	1	0.59
		Group 5 (*n* = 0)	Group 5 (*n* = 1)			
		Group 6—26.53% (*n* = 177)	Group 6—24.56% (*n* = 83)	Pearson Chi square	1	0.50
		Group 7—11.39% (*n* = 76)	Group 7—10.36% (*n* = 35)	Pearson Chi square	1	0.62

Variable	Total (*n*) assessed: ED (*n* = 660), Non‐ED (*n* = 392)	ED cohort	Non‐ED cohort	Test	df	Sig (2‐sided *p* value)
Clinical characteristics						
Mean log(Fibroscan LSM)	ED: *n* = 623 Non‐ED: *n* = 342	0.73 (SD = 0.15)	0.71 (SD = 0.18)	Independent sample *t*‐test	963	0.25 (mean difference 0.013)
Controlled attenuation parameter (CAP)	ED: *n* = 581 Non‐ED: *n* = 327	244.98 dB/m (SD = 57.67)	236.87 dB/m (SD = 57.78)	Independent sample *t*‐test	906	0.04 (mean difference 8.1)
≥ F2 (LSM > 7.0)	ED: *n* = 623 Non‐ED: *n* = 342	16.37% (*n* = 102)	17.25% (*n* = 59)	Pearson Chi square	1	0.72
≥ F4 (LSM > 12.5)	ED: *n* = 623 Non‐ED: *n* = 342	2.09% (*n* = 13)	4.68% (*n* = 16)	Fisher's exact test	1	0.03
Mean log(HBV DNA)	ED: *n* = 660 Non‐ED: *n* = 392	2.08 (SD = 1.69)	2.78 (SD = 1.81)	Independent sample *t*‐test	1043	< 0.001 (mean difference 0.71)
HBV DNA ≥ 2000 IU/mL	ED: *n* = 660 Non‐ED: *n* = 392	20.45% (*n* = 135)	30.87% (*n* = 121)	Pearson Chi square	1	< 0.001
HBV DNA ≥ 20 000 IU/mL	ED: *n* = 660 Non‐ED: *n* = 392	7.88% (*n* = 52)	12.24% (*n* = 48)	Pearson Chi square	**1**	0.02
Treated within the censor dates	ED: *n* = 660 Non‐ED: *n* = 392	14.39% (*n* = 95)	36.5% (*n* = 143)	Pearson Chi square	1	< 0.001

Variable	Exp (*B*)/Odds ratio	95% CI	Sig. (*p* value)
Multivariate analysis			
Age	0.97	0.96–0.98	< 0.001
Gender (male)	0.79	0.57–1.1	0.16
UK‐born	2.56	0.72–9.25	0.14
English as 1st language	0.51	0.19–1.37	0.18
HBV DNA > 2000 IU/mL	1.82	1.28–2.60	< 0.001
Log (Fibroscan LSM)	0.79	0.28–2.27	0.67

*Note:* Multivariate analysis was performed using binary logistic regression; Exp (*B*) values represent the odds ratio of a non‐ED rather than ED testing referral in equivalent recruitment periods of referral to regional hubs.

**FIGURE 3:**
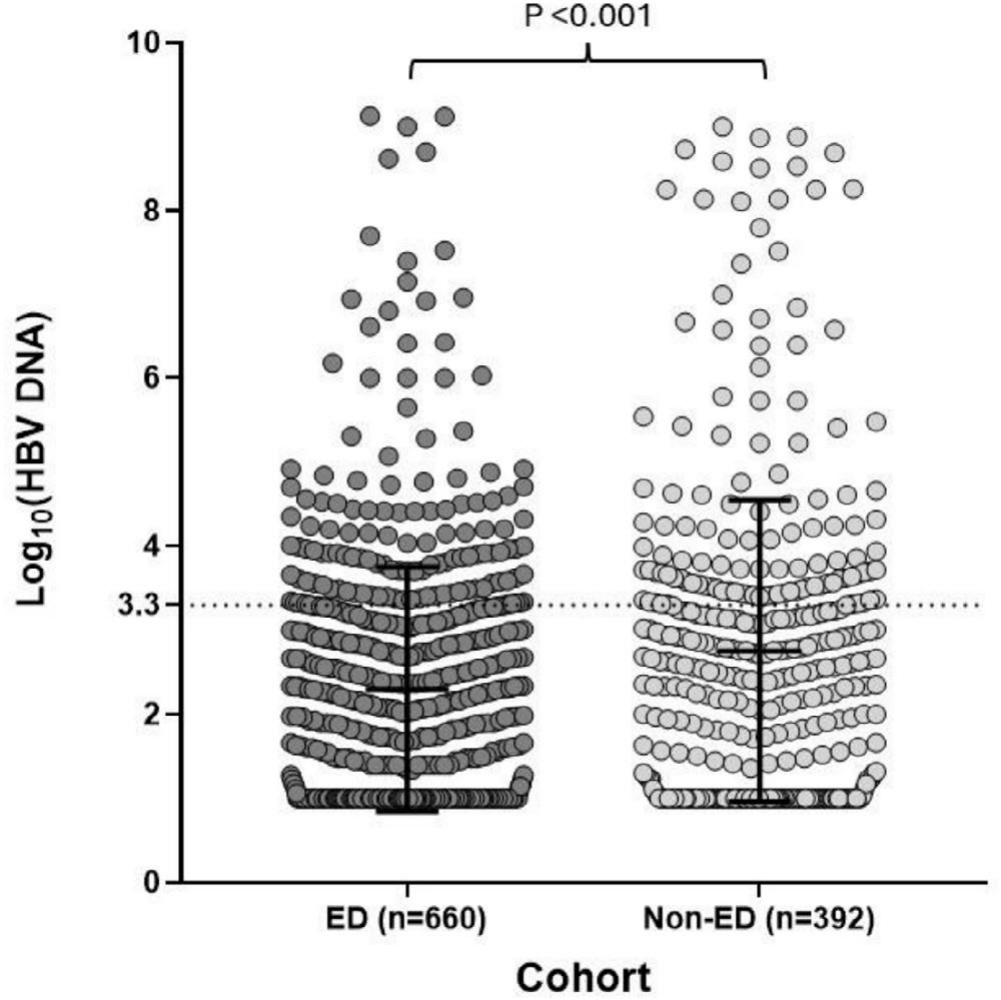
Comparison of _Log_(HBV DNA) levels between individuals from the ED and non‐ED cohorts. The dotted line represents an HBV DNA level of 2000 IU/mL indicating at least moderate viraemia and a possible indication for treatment. The black solid line and whiskers show mean and standard deviation (SD). The *p*‐value was calculated by using independent *t*‐test; a higher mean _Log_(HBV DNA) level was found in the ‘non‐ED’ group.

Mean _Log_(LSM) was not significantly different between ED and non‐ED cohorts on univariate analysis (0.73 ED vs. 0.71 non‐ED, NS). In the ED group, there was a higher average controlled attenuation parameter (CAP) score, suggesting patients in the ED cohort may also be more likely to have hepatic steatosis (245 dB/m ED vs. 237 dB/m non‐ED, 2‐sided *t*‐test, *p* value 0.04). Individuals presenting through the ED pathway had a lower HBV viral load (mean _Log_HBVDNA 2.08 ED vs. 2.78 non‐ED, two‐tailed *t*‐test, *p* value < 0.001) (Figure 3). This likely impacted the proportion of patients from each group who were commenced on nucleoside analogues during the observation period, which was also lower in the ED group (14.39% ED vs. 36.5% non‐ED, two‐sided Chi‐Squared test, *p* value < 0.001).

**FIGURE 4:**
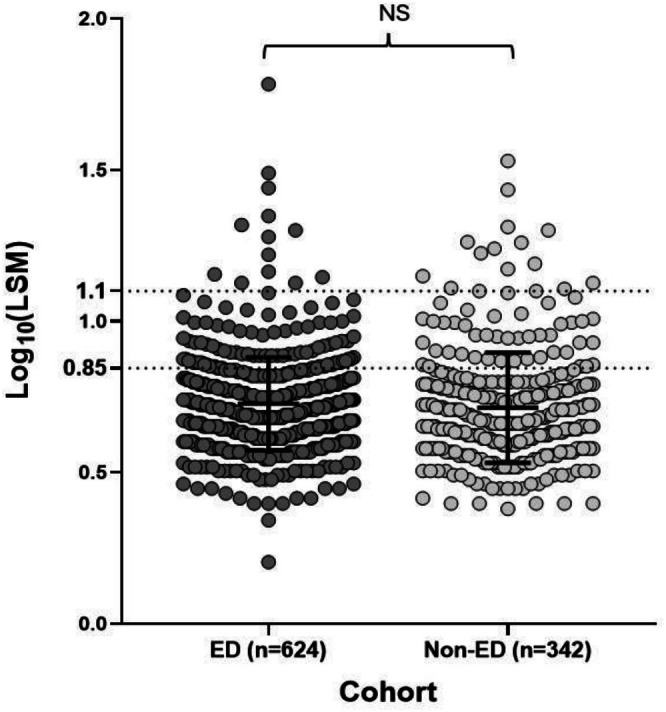
Comparison of FibroScan liver stiffness measurements (LSM) in individuals from the ED and non‐ED cohorts in North Central London and Southwest London. LSM values are shown on a logarithmic scale. The dotted lines indicate the LSM thresholds for F2 fibrosis (7.0 kPa) and F4 fibrosis (12.5 kPa). The solid black line and whiskers show the mean and standard deviation (SD). Mean _Log_(LSM) values did not differ significantly between cohorts (NS); the *p*‐value was calculated using an independent *t*‐test.

A binary logistic regression multivariate analysis was performed to identify characteristics associated with presentation via the non‐ED route (reference category: ED route). Higher viral load (OR 1.82; 95% CI 1.28–2.60) was significantly associated with diagnosis via the non‐ED route; conversely, older age was associated with the ED route of presentation (OR 0.97 per year increase) (Table 2). LSM scores as a continuous variable did not predict non‐ED presentation (Figure 4).

**FIGURE 5:**
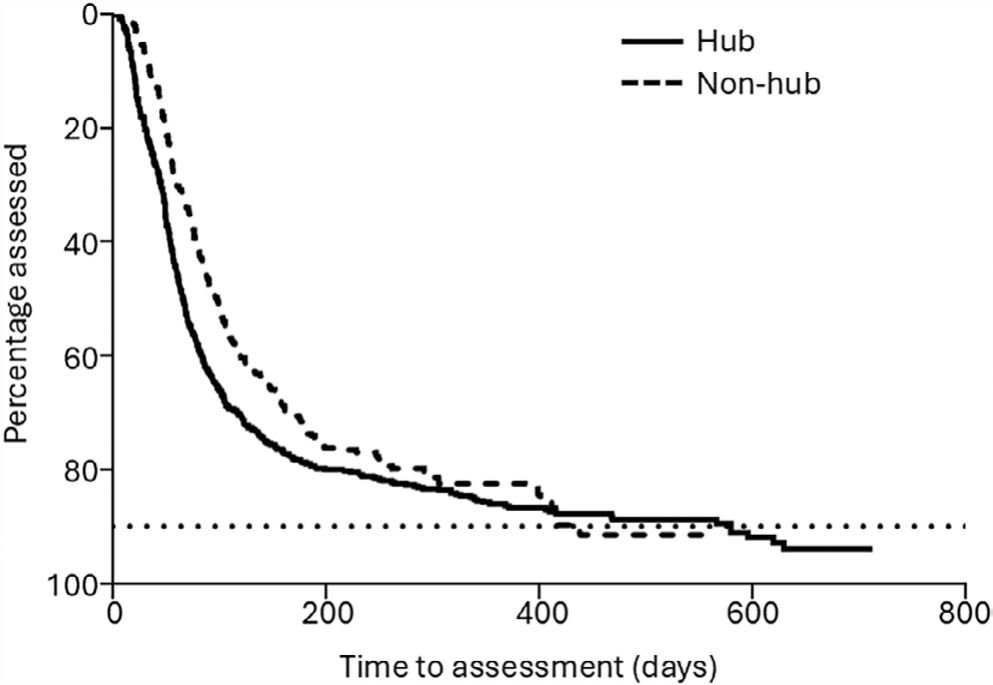
Comparison of time to assessment in the ED testing cohort in those diagnosed and assessed in the same healthcare organisation (hub) and those diagnosed at an ED in a different healthcare organisation (non‐hub) before assessment at the hub site. The horizontal dotted line represents 90% of patients assessed in each group. Log‐rank Mantel Cox test of difference *p* < 0.002, Gehan‐Breslow‐Wilcoxon test *p* < 0.0001.

The mean time from lab notification of the viral hepatitis team to assessment was 90 days (SD = 82.3) in the ED cohort and median time from notification to assessment was 65 days (IQR 41.43–107.64). On Cox regression multivariate analysis, only assessment at the same ED site of diagnosis increased the likelihood of assessment over time (HR 1.57, 95% CI 1.33 to 1.87, *p* value < 0.001) (Table 3). By one year, the proportions of referred patients assessed at all sites were the same (Figure 5).

**TABLE 3:** Cox regression multivariate analysis of factors affecting time from notification of positive HBsAg result to assessment in the ED cohort.

Variable	Exp (B)	(95% CI)	Sig. (*p* value)
Planned assessment at same organisation	**1.57**	**(1.33–1.87)**	**< 0.001**
Aware of diagnosis	1.03	(0.87–1.23)	0.72
Age	1.0	(0.99–1.01)	0.77
Gender (Male)	0.95	(0.8–1.13)	0.55
UK born	0.89	(0.49–1.61)	0.71
English 1st language	0.79	(0.54–1.17)	0.24
Drinking > 14 units per week	0.82	(0.59–1.14)	0.23

*Note:* ‘Planned assessment at same organisation’ describes patients diagnosed and assessed at an ED and viral hepatitis clinic within the same healthcare organisation.

## 4 Discussion

ED opt‐out testing for hepatitis B increased new assessments by 107% across the seven sites during the observation period. The majority were unaware of their diagnosis and 60.2% had never been assessed by specialist services, reflecting the large numbers of adults living with undiagnosed or unassessed hepatitis B infection. Over 90% of patients in the ED cohort were born in countries with medium to high HBV prevalence. As per national guidance, this should trigger targeted opportunistic testing in primary and secondary care and onward referral for specialist care.

Only 10% of patients with a positive HBsAg result could not be contacted. In the 90% contacted, the rate of subsequent engagement for assessment was very high (97%) but it took a mean time of 3 months from the original diagnosis to assessment and more than 400 days to assess > 90% of patients. A recent interim UKHSA evaluation of the ED testing programme, for example, reported linkage to care as 38%, but this was defined using clinic attendance within 4 weeks of a positive test, highlighting the importance of selecting a realistic cut‐off date when evaluating linkage to care [18].

Significant differences in age and ethnicity between ED and non‐ED cohorts suggest ED opt‐out testing is reaching people who are less likely to be tested in existing pathways. Patients diagnosed through non‐ED pathways are more likely to have cirrhosis and higher viral loads (despite a younger average age) suggesting a substantial proportion of non‐ED diagnosis is through reactive testing (e.g., after abnormal liver function tests or imaging) rather than targeted testing (e.g., country of birth). Diagnosis through ED testing at an earlier stage of liver disease may result in greater benefit through preventative treatment and HCC surveillance. It is noteworthy that new European Association for the Study of the Liver (EASL) guidelines issued in May 2025 would mean more patients would now meet treatment thresholds than in this evaluation period [26]. Although the number of individuals with HBV/HDV coinfection in the ED cohort was small (*n* = 7) identifying these individuals and linking them to care is crucial given their accelerated disease course and greater risk of HCC.

Over one third of patients in the ED cohort who were not under follow‐up were already aware of their diagnosis but were either lost to follow‐up or had never previously been assessed in specialist services. Prior to national guidance (NICE CG165 2013) which recommended lifelong specialist follow‐up for hepatitis B infection, some patients were told they could be discharged back to primary care because they are ‘inactive carriers’. These data suggest there is a strong case for a re‐engagement programme to bring patients back under specialist care, facilitated by national patient‐identifiable sentinel surveillance data.

An analysis of socioeconomic status and testing in these cohorts was beyond the scope of this study. A further limitation of this study is that we did not assess the engagement outcomes in the small numbers of individuals who were diagnosed through ED testing but referred to a service closer to home, nor have outcomes of those with equivocal test results that were due to be retested in primary care been fully determined.

We found that subsequent to diagnosis, planned assessment within the same organisation as the diagnosing ED site was the only factor that significantly increased the likelihood of assessment over time. Whilst provision of hub clinics was necessary to offer timely specialist assessment of new patients (in the context of much longer waiting lists for general hepatology clinics locally), the requirement of patients to travel to non‐local hospital sites for assessment may have delayed assessment. This highlights the importance of not only service capacity but also accessibility in reducing barriers to engagement.

The large numbers of people lost to follow‐up who were detected in the programme indicate current service capacity for maintaining engagement is already critically limited. The doubling of new referrals through this highly successful case‐finding programme will impact this further. To prevent deaths from liver cancer, expanded services redesigned by those living with hepatitis B infection are needed so that both new and existing patients can access and benefit from treatment and monitoring.

## Author Contributions

All authors were involved with the conception of the manuscript. Jennifer J. Plunkett and Basil Ahmad wrote the first draft. Douglas Macdonald and Daniel Forton provided critical revisions. All authors provided revisions and approved the final manuscript. Douglas Macdonald is guarantor.

## Funding

The authors have nothing to report.

## Ethics Statement

This manuscript has been reviewed by the Group Deputy Director of R&I, St George's and Epsom and St Helier hospitals group. This review has confirmed that this work constitutes a service evaluation under Health Research Authority (HRA) definitions. As such, and in accordance with national guidelines, this project does not require NHS Research Ethics Committee (REC) or HRA approval as it does not constitute a research study.

## Conflicts of Interest

Rachel Hill‐Tout is national lead for the ED BBV opt‐out testing programme and has received speaker fees from Gilead Sciences. Philippa C. Matthews has received funding support from GSK for a doctoral fellow in her research team and to support a collaboration with the UK Health Informatics Collaborative for Viral Hepatitis and Liver Disease. Helen Boothman currently works for GSK but worked for the St George's University Hospitals Foundation NHS Trust at the time of this evaluation. No financial support was received for this manuscript.

## Data Availability

Data used in this manuscript can be accessed upon reasonable request by contacting the manuscript guarantor (douglasmacdonald@nhs.net), the purpose of accessing the data should be made clear in the request. De‐identified individual participant data will be available from the point of submission with no end date. No other documents will be available.
